# Coupling to a cancer-selective heparan-sulfate-targeted branched peptide can by-pass breast cancer cell resistance to methotrexate

**DOI:** 10.18632/oncotarget.19056

**Published:** 2017-07-06

**Authors:** Lorenzo Depau, Jlenia Brunetti, Chiara Falciani, Silvia Scali, Giulia Riolo, Elisabetta Mandarini, Alessandro Pini, Luisa Bracci

**Affiliations:** ^1^ Department of Medical Biotechnologies, University of Siena, 53100 Siena, Italy

**Keywords:** peptides, methotrexate, drug-resistance, heparan sulfate

## Abstract

Cancer-selective tetra-branched peptides, named NT4, can be coupled to different functional units for cancer cell imaging or therapy. NT4 peptides specifically bind to lipoprotein receptor-related proteins (LRP) receptors and to heparan sulfate chains on membrane proteoglycans and can be efficiently internalized by cancer cells expressing these membrane targets. Since binding and internalization of NT4 peptides is mediated by specific NT4 receptors on cancer cell membranes and this may allow drug resistance produced by drug membrane transporters to be by-passed, we tested the ability of drug-armed NT4 to by-pass drug resistance in cancer cell lines.

We found that MTX-conjugated NT4 allows drug resistance to be by-passed in MTX-resistant human breast cancer cells lacking expression of folate reduced carrier. NT4 peptides appear to be extremely promising cancer-selective targeting agents that can be exploited as theranostics in personalized oncological applications.

## INTRODUCTION

The effectiveness of any cancer chemotherapy is profoundly limited by drug resistance. Drug resistance can be divided into two main categories: intrinsic and acquired [1]. In intrinsic resistance, cancer cells respond weakly or not at all to the drug from the beginning of treatment due to pre-existing factors. In acquired resistance, cancer cells, which initially respond to the drug, become progressively resistant and cease to respond due to many different therapy-induced adaptive mechanisms. Though extremely problematic, intrinsic drug resistance can be circumvented by alternative medical options. Acquired resistance is even more problematic, because it is very difficult to predict and sometimes involves simultaneous resistance to many different chemotherapy drugs, often resulting in multi-drug-resistant (MDR) tumor variants [1, 2] that call for personalized therapy.

Intrinsic and acquired drug resistance both arise from genetic and epigenetic modification of cancer cells. Drug resistance can be induced in cancer cells by different adaptive molecular mechanisms, including modification of drug targets, modification of the metabolic pathway responsible for drug activation or activity, modification of drug influx or efflux, and activation of savage pathways. In any case, drug resistance implies modifications to the function and/or expression of enzymes or membrane transporters. MDR is often associated with overexpression of membrane-active transporters responsible for drug extrusion out of the cell [3, 4].

Different mechanisms account for drug internalization by cancer cells. Hydrophobic molecules can be passively transported across cell membranes, which implies low selectivity and possibly toxicity toward healthy cells, as well as a decrease in active drug concentration at the tumor site. Specific transporters are responsible for cell internalization of important cancer chemotherapy drugs that do not passively cross cell membranes, like those belonging to the class of antifolates, such as methotrexate (MTX) and aminopterin. By closely mimicking folate structure, antifolates use folate-specific membrane transporters and receptors for cell internalization [5]. Two membrane facilitative carriers, reduced folate carrier (RFC) and proton-coupled folate transporter (PCFT), and three high affinity folate receptor glycoproteins (FRα, FRβ and FRγ), all of which are widely expressed by normal tissues, account for antifolate cell internalization. RFC is the major transport system for antifolate drugs into cancer cells.

Once internalized, folate analogs are polyglutamylated by folylpolyglutamate synthetase, which produces stable folate polyglutamates that are retained in the cell, since they are poor substrates for folate export transporters. The cytotoxic effect of antifolates is exerted by inhibition of dihydrofolate reductase (DHFR) and/or thymidylate synthase (TS) and blockade of key folate-dependent enzymes in the interconnected biosynthetic pathways of purine, thymidylate and some amino acids, like methionine, ultimately resulting in inhibition of DNA replication and cell death [5–8]. Adverse effects associated with antifolates include severe damage to normal cells and organs, narrow therapeutic index, poor selectivity for neoplastic cells, and cancer cell resistance to the drugs.

Intrinsic and acquired resistance to antifolates is unfortunately quite a common event and has dramatic consequences on cancer therapy efficiency. Resistance to antifolates is frequently caused by impaired drug membrane transport, caused by low expression or mutations in RFC. Alternative or coexisting causes of folate resistance are mutations or aberrant expression of enzymes of folate metabolism, like folylpolyglutamate synthetase, TS and DHFR [5–9]. Overexpression of MDR efflux transporters can also account for antifolate resistance, although it is important to note that no induced overexpression of MDR transporters has ever been reported in cancer cell lines selected for antifolate resistance [6].

In previous studies we investigated the cancer selectivity of tetra-branched peptides containing the sequence of human neurotensin, which we named NT4. We developed theranostic molecules for tumor imaging or therapy by coupling NT4 to different functional units or liposomes. NT4 peptides bind with high selectivity to cells and tissues of different human cancers, such as colorectal cancer, pancreas adenocarcinoma and urinary bladder cancer, and can efficiently and selectively deliver drugs or liposomes for cancer therapy or carry tracers for tumor imaging. Using NT4 conjugated to MTX or 5-floro-2′deoxyuridine, we obtained a significant reduction in tumor growth in mice -60% and 50% respectively-, compared with that obtained with equal amounts of unconjugated drug [10–14]. More recently, we found that conjugation of paclitaxel to NT4 led to increased therapeutic activity of the drug in an orthotopic model of breast cancer in mice, producing tumor regression which was not achieved with unconjugated paclitaxel in identical experimental conditions [15].

The cancer selectivity of NT4 peptides is much higher than that of native monomeric neurotensin, which unlike NT4 does not discriminate between healthy human tissues and cancer [10, 12, 14]. We demonstrated that the branched structure gives NT4 the ability to bind sulfated glycosaminoglycan (GAG) chains of membrane heparan sulfate proteoglycans (HSPG), as well as different endocytic receptors of the low density lipoprotein receptor (LDLR) family, like LRP1 and LRP6 [16, 17]. LDLR are already known to share many heparin-binding ligands with HSPG and both families include potential druggable tumor markers involved in cancer biology [18–21].

Systematic modification of the neurotensin sequence in NT4 peptides led to identification of a positively-charged multimeric motif that mediates interaction with heparin and endocytic receptors [16]. By binding to their selective targets on cancer cell membranes, NT4 peptides inhibit cancer cell adhesion and migration on different extra cellular matrix proteins, dramatically affecting the directionality and polarity of cell movement [17]. The extremely high cancer selectivity that can be achieved by targeting sulfated GAGs with NT4 [16, 17] confirms the already reported overexpression of these membrane receptors in cancer cells [18, 19, 22–25], which renders them potential tumor markers and cancer selective targets and suggests that NT4 peptides may indeed be selective targeting agents for many different cancers.

In previous studies we demonstrate that NT4 peptides are efficiently internalized by different cancer cells and that internalization is strictly dependent on NT4 membrane targets [10, 12, 14]. This raised the question of whether by binding to different membrane receptors on cancer cells, and switching to a different internalization mechanism, drug-armed NT4 may by-pass drug resistance mediated by specific drug membrane transporters.

Since cancer cell resistance to antifolates is often caused by mutation or down-regulation of membrane folate carriers, we tested whether conjugation to NT4 might by-pass cancer cell resistance to MTX. Cytotoxicity of MTX was tested on several human cancer cell lines. All proved sensitive to MTX with comparable EC50, with the exception of the human breast cancer cell line MDA-MB 231, which was less sensitive to MTX than the others, including the breast cancer cell line MCF-7. We then compared the internalization and cytotoxicity of free and NT4-conjugated MTX in two breast cancer cell lines: MTX-resistant MDA-MB 231 and MTX-sensitive MCF-7. We found that by coupling MTX to NT4, cancer cell drug resistance to MTX could be by-passed.

## RESULTS

### Synthesis of NT4-MTX

**Figure 1:**
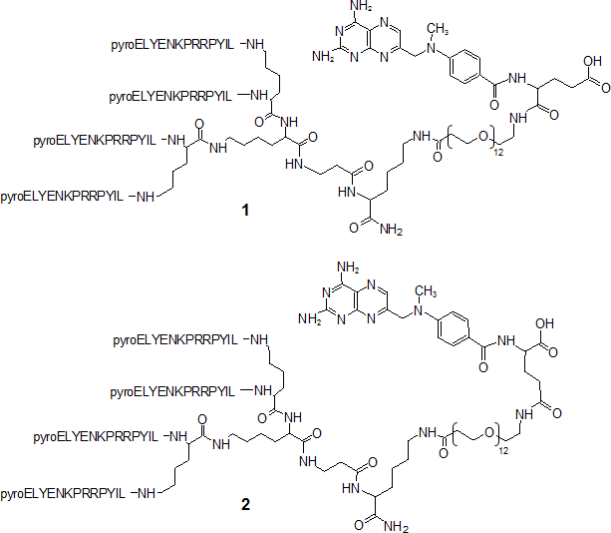
Structures of NT4 peptide conjugated with methotrexate using Cα of Glu, (**1**) NT4-α-MTX and Cγ of Glu, (**2**) NT4-γ-MTX.

NT4 peptide was synthesized by the solid-phase method and conjugated with methotrexate. MTX could be coupled to NT4 using the two different carboxylic groups, α and γ of the glutamic acid, giving two regioisomers that could have different efficacy. We prepared both isomers by linking MTX to NT4 through an amide bond originating from the free carboxyl group in α or γ position of the drug and the amine group of the peptide (Figure 1). Identity and purity of the final products, NT4-α-MTX and NT4-γ-MTX, were confirmed by analytical reverse-phase chromatography on a Jupiter C18 column and mass spectrometry.

### Screening of human cancer cell line for MTX sensitivity

In order to select MTX-resistant cancer cells, MTX cytotoxicity was tested in different human cancer cell lines (Table 1). All screened cancer cells proved sensitive to MTX with comparable EC50 (about 10^−8^M) with the sole exception of human breast cancer cell line MDA-MB 231, which was less sensitive to MTX (EC50 5.06 × 10^−7^ M). The two human breast cancer cell lines, MDA-MB 231 and MCF-7, which had different sensitivity to MTX, were then selected to test NT4-MTX for its ability to circumvent MTX resistance.

**Table 1:** EC50 of MTX on several human cancer cell lines

	Cell line	EC50 of MTX
MCF-7	human breast adenocarcinoma	2.58 × 10^−8^M
MDA-MB 231	human breast adenocarcinoma	5.06 × 10^−7^M
T-24	human bladder carcinoma	8.07 × 10^−8^M
PANC-1	human pancreatic carcinoma	2.34 × 10^−8^M
HT29	human colon adenocarcinoma	6.09 × 10^−8^M
A375	human melanoma	3.37 × 10^−8^M
HT-1376	human bladder carcinoma	1.71 × 10^−7^M
OVCAR-3	human ovarian carcinoma	8.95 × 10^−8^M

### Binding and internalization of NT4 in MTX-sensitive and MTX-resistant human breast cancer cell lines

**Figure 2:**
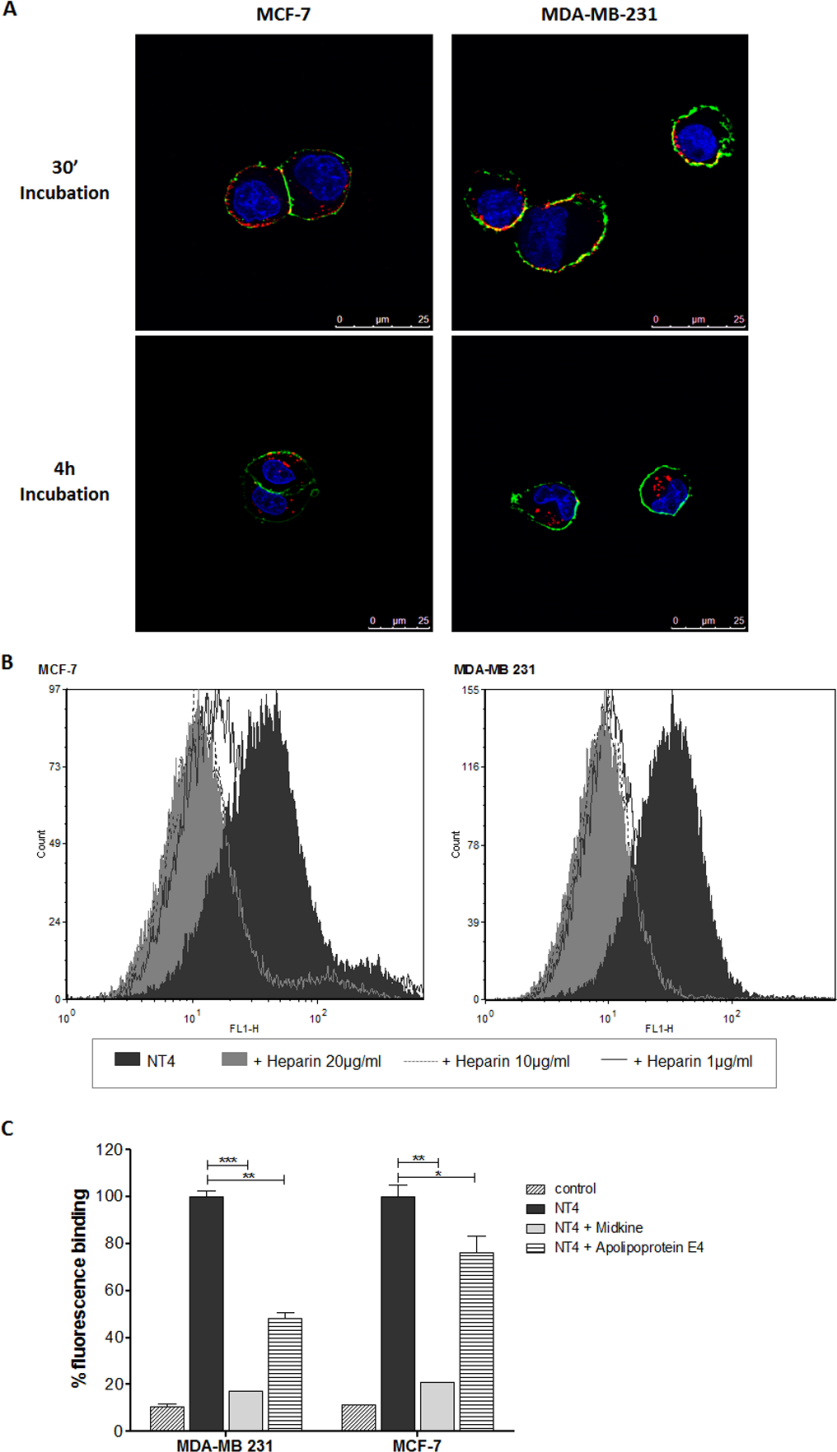
NT4 binding to MCF-7 and MDA-MB 231 (**A**) Binding (30’ incubation) and internalization (4 h incubation) of NT4 (red) in MCF-7 (left panel) and MDA-MB 231 cells (right panel) analyzed by confocal microscopy. Nuclei were stained with DAPI (blue) and plasma membrane was stained with wheat germ agglutinin (green). (**B** and **C**) Flow cytometry analysis of NT4 binding to MCF-7 and MDA-MB 231 cells and its inhibition by different concentrations of heparin (B) or by heparin binding proteins Apolipoprotein E4 and Midkine (C). Florescence of cells without NT4 is taken as control. Data presented as mean ± SD. *p < 0.05, **p < 0.01, ***p < 0.001 calculated using two-tailed Student *t*-test using GraphPad Prism 5.03 software.

Binding and cell internalization of NT4 peptide, conjugated with biotin and traced by streptavidin-FITC, was tested in MTX-sensitive MCF-7 and MTX-resistant MDA-MB 231 human breast cancer cells and proved identical in the two cell lines (Figure 2A). In a previous study we demonstrated that NT4 binding to its membrane targets on cancer cells is inhibited by heparin, which can also completely abolish NT4 selectivity toward human cancer tissues [16]. Flow cytometry analysis showed that binding of NT4 to MCF-7 and MDA-MB 231 cells was inhibited by heparin (Figure 2B) and by heparin binding proteins like Apolipoprotein E4 and Midkine (Figure 2C). This confirmed that binding of NT4 to MCF-7 and MDA-MB 231 is specific and mediated by the same NT4 membrane receptors.

### MTX transporters in MTX-sensitive and MTX-resistant human breast cancer cell lines

**Figure 3:**
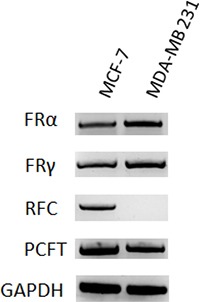
Folate transporters in MTX-sensitive and MTX-resistant human breast cancer cell lines Gene expression of human FR α, FR γ, RFC and PCFT in MCF-7 and MDA-MB 231 cell lines by RT-PCR. Human GAPDH was used as experimental control.

Transport of folate compounds into mammalian cells can occur via receptor-mediated or carrier-mediated mechanisms. Three genetically distinct and functionally diverse transport systems are involved in membrane transport of folates into mammalian cells, including RFC, PCFT, and folate receptors. RFC is the major systemic transport system for antifolate drugs in cancer cells [26–29]. Modified expression of folate membrane transporter or receptors is a common event leading to cell resistance to antifolates [5–9]. Given the different sensitivity of the two breast cancer cell lines to MTX, we analyzed gene expression of human RFC, PCFT and the three major isoforms α, β, and γ of FR in MCF-7 and MDA-MB 231 cells by RT-PCR (Figure 3). The breast cancer cell line MCF-7 and MDA-MB 231 displayed similar expression of PCFT, FR α and FR γ, whereas MDA-MB 231 cells lacked expression of RFC, which was instead expressed in MCF-7. As already reported, FR β was not detected in either cell line (data not shown) [30]. The lack of RFC could account for the higher resistance of MDA-MB 231 to MTX cytotoxicity.

### Drug influx and efflux in MTX-sensitive and MTX-resistant human breast cancer cell lines

**Figure 4:**
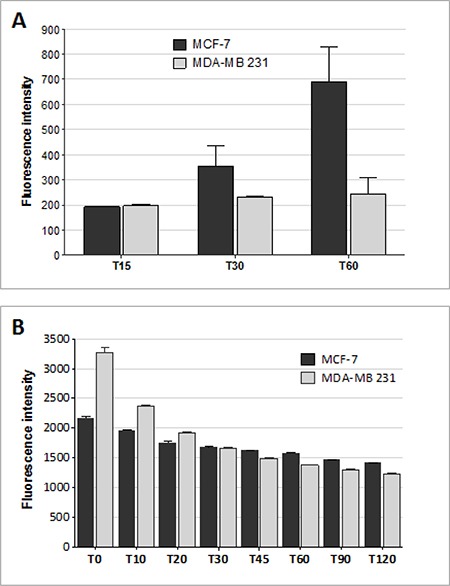
Drug influx and efflux in MTX-sensitive and MTX-resistant human breast cancer cell lines (**A**) Fluorescein-MTX internalization in MCF-7 and MDA-MB 231 cells after 15, 30 and 60 min of incubation, as measured by flow cytometry. Data presented as median ± SD. (**B**) Rhodamine 123 signal on MCF-7 and MDA-MB 231 cells measured by flow cytometry. Cells were incubated for 10 minutes with Rho 123 and washed. Green fluoresce was monitored immediately (T0) and after 10, 20, 30, 45, 60, 90 and 120 minutes by FACS Canto II. Data presented as median ± SD.

Internalization of MTX conjugated with fluorescein (fluo-MTX) was monitored in MTX-sensitive and MTX-resistant human breast cancer cell lines by flow cytometry (Figure 4A). MCF-7 and MDA-MB 231 cells were incubated for 15, 30 and 60 min with 10 μM fluo-MTX and the green fluorescent signal was analyzed in 10,000 cells. Fluo-MTX was more efficiently internalized in MCF-7 cells than in MDA-MB 231. As shown in Figure 4A, fluo-MTX signal in MCF-7 was 2.7-fold higher than in MDA-MB 231 after 1 hour of incubation.

Since the amount of drug retained in a cell is the resultant of internalization and export rates, we compared the general drug efflux ability of MDA-MB 231 and MCF-7 cells. Rhodamine 123 (Rho 123), a member of the rhodamine family of fluorescent dyes, has been used as a tracer for efflux membrane transport [31, 32]. Rho 123 is lipophilic and can be transported passively inside cells, although its internalization can also be at least partially mediated by membrane transporters. Rho 123 export out of cells is actively mediated by ABC transporters. It has been used for analyzing active cell drug export mechanisms and related MDR [33].

The transporter-mediated efflux of Rho 123 was measured by following Rho 123 green signal in MCF-7 and MDA-MB 231. Cells were incubated with 1 μM Rho 123 for 10 minutes. After washing, fluorescence was monitored by flow cytometry (Figure 4B). Despite an initially higher fluorescent signal in MDA-MB 231 at time 0, the signal decrease at each time interval was similar in MDA-MB 231 and MCF-7, indicating that drug efflux was comparable in the two cell lines.

### Cytotoxicity of free and NT4-conjugated MTX in human breast cancer cell lines

**Figure 5:**
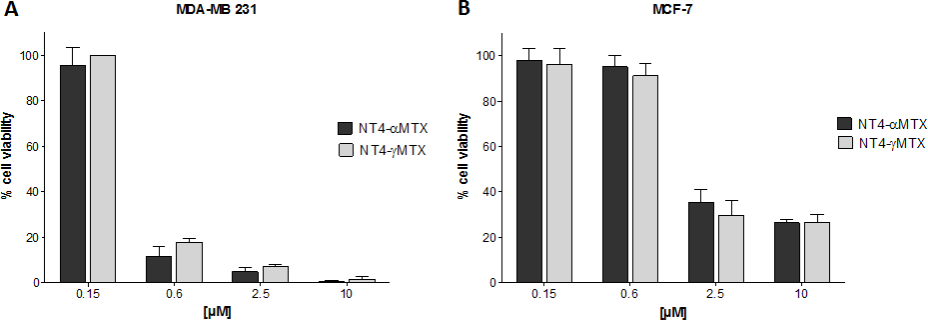
Cytotoxicity of NT4-α-MTX (dark grey) and NT4-γ-MTX (light grey) in MTX-resistant MDA-MB 231 (**A**) and MTX-sensitive MCF-7 (**B**) human breast cancer cell lines. Data presented as mean ± SD.

The cytotoxicity of NT4-α-MTX and NT4-γ-MTX was tested in human MTX-sensitive MCF-7 and MTX-resistant MDA-MB 231 cells (Figure 5). The regiochemistry of the conjugation of NT4 to MTX proved to have no impact on the overall cytotoxic effect, since the two regioisomers, NT4-α-MTX and NT4-γ-MTX, were completely identical in this test.

**Figure 6:**
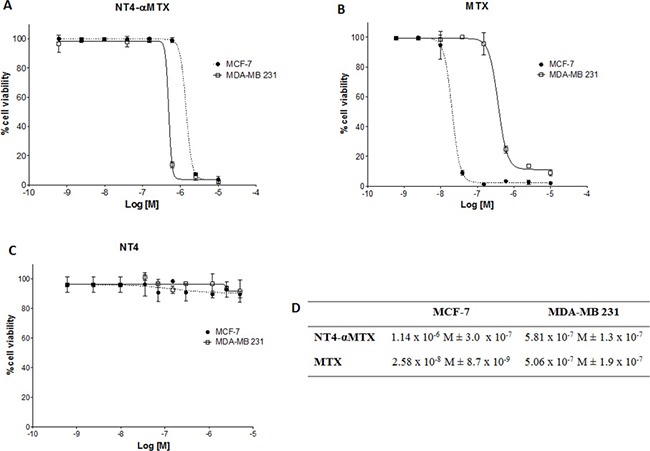
Cytotoxicity of NT4-α-MTX in MTX-sensitive and MTX-resistant human breast cancer cell lines; NT4-α-MTX (**A**) free MTX (**B**) and unconjugated NT4 (**C**) in MCF-7 and MDA-MB 231 cell lines. (**D**) EC50 of NT4-α-MTX and free MTX in MCF-7 and MDA-MB 231 cells. Data presented as mean of six experiments ± SD.

Cellular toxicity of NT4-α-MTX was then compared to that of the corresponding free drug in MDA-MB 231 and MCF-7 cells. MCF-7 cells were more sensitive than MDA-MB 231 to unconjugated MTX (EC50 2.58 × 10^−8^ M and 5.06 × 10^−7^ M, respectively). Nonetheless, NT4-α-MTX was slightly more effective in MDA-MB 231 than in MCF-7 (EC50 5.81 × 10^−7^ M and 1.14 × 10^−6^ M, respectively) (Figure 6). EC50 of NT4-α-MTX in MCF-7 and MDA-MB 231 was in line with that previously measured by us in different cell lines [12, 14] and also with the EC50 of different chemotherapy drugs, when conjugated to NT4 [11, 14]. No cytotoxic effect was measured using unconjugated NT4 at the same concentrations (Figure 6C).

**Figure 7:**
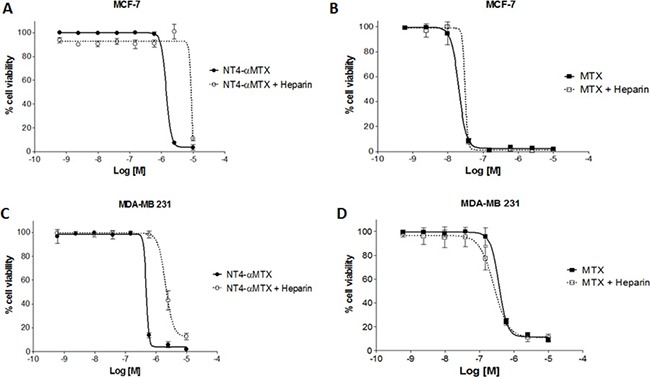
Cytotoxicity of NT4-α-MTX in human breast cancer cell lines, with and without heparin; NT4-α-MTX and free-MTX in MCF-7 (**A** and **B**) and MDA-MB 231 (**C** and **D**).

To confirm specificity of NT4-α-MTX cytotoxicity in MTX-sensitive and MTX-resistant cells, we analyzed the effect of heparin on the efficacy of NT4-α-MTX with respect to the free drug. MCF-7 and MDA-MB 231 cells were exposed to different concentration of NT4-α-MTX or MTX in the presence of 1 μM heparin, which had previously been confirmed as not affecting cancer cell growth (not shown), and cell growth was analyzed after 6 days of incubation (Figure 7). Heparin significantly decreased cytotoxicity of NT4-α-MTX in both cell lines, whereas it did not affect cytotoxicity of unconjugated MTX.

## DISCUSSION

For years tumor-targeted therapy has been considered a necessary development in cancer therapy to overcome the toxicity of classical chemotherapy drugs, the low selectivity of which inevitably damages healthy tissues and systems. The basis of tumor-targeted agents resides in cancer-specific antigens and/or cell pathways, the targeting of which may allow selective killing of cancer cells by direct or indirect mechanisms. Unfortunately no universal specific tumor target, selectively expressed by all tumors at any differentiation grade, has yet been identified. The efficiency of tumor targeting agents is therefore linked to preliminary identification of their specific target in a given tumor and a given patient. Thus cancer-selective targeting agents with double diagnostic and therapeutic use, i.e. theranostics, are very convenient for effective personalized therapy.

In a theranostic oncological approach, specific targeting agents, like antibodies, peptides or nanoparticles, are used as cancer-selective carriers for delivery of drugs, dyes or radioisotopes for *in vitro* and *in vivo* imaging, which may be followed or accompanied by cancer cell killing. For such an approach, molecular targets overexpressed on cancer cell membranes are particularly attractive since they may allow selective binding of the targeting agent to cancer cells. Binding of the theranostic agent to membrane targets may suffice for cancer cell imaging, whereas cell internalization is usually necessary for therapy. The internalization of a drug-armed carrier after binding to a cancer cell membrane target can have the important advantage of preserving cells that do not express the membrane target. Moreover, drug internalization through a target-mediated mechanism can overcome drug resistance when this is mediated by modifications of membrane drug transporters.

Since the first approval of the tumor-targeted monoclonal antibody Rituximab by the FDA in 1997, several tumor-selective agents have been approved. The vast majority of the drugs presently available for targeted therapy are antibodies recognizing membrane antigens, however small molecules interfering with tumor biological pathways, like the kinase inhibitors, are also in clinical use [34]. More recently, peptides have also been proposed as cancer-selective targeting agents, and these may combine some advantages of small molecules and antibodies [35]. Peptides have small molecular mass, they are obtained by chemical synthesis and can easily be conjugated to drugs with a favorable carrier-drug ratio, which is the main limit of antibody-drug conjugate activity [36], and can have cell selectivity similar to antibodies [35].

The finding that peptides synthesized in branched form are resistant to circulating proteases and peptidases [37] and that they can easily be conjugated to different functional units without interfering with their biological action [10, 11] paved the way for their use as cancer theranostics. In previous papers we investigated cancer-selective tetra-branched peptides, named NT4, which can be coupled to different functional units for cancer cell imaging or therapy. NT4 peptides bind to LRP receptors and to heparan sulfate chains on membrane proteoglycans and can be efficiently internalized by cancer cells expressing these membrane targets [10–17].

Since our demonstration that cell internalization of NT4 peptides conjugated to functional units is mediated by peptide-specific membrane receptors overexpressed by cancer cells [10–12, 15, 16], we set out to check whether NT4 could overcome cancer cell drug resistance by mediating drug internalization into cells by a drug-transporter-independent mechanism.

We selected MTX as carried drug because cancer cell resistance to antifolate is often caused by modification in activity or expression of membrane folate transporters. We tested several different human cancer cell lines for sensitivity to MTX and found similar EC50 for all except the human breast cancer cell line MDA-MB 231, which was about ten times less sensitive.

In order to test whether cell resistance to MTX could be overcome by conjugating the drug with NT4, we compared binding, internalization and cytotoxicity of free and carrier-bound MTX on two breast cancer cell lines, MTX-resistant MDA-MB 231 and MTX sensitive MCF-7. The two cell lines proved to have similar ability to bind and internalize NT4 peptides, as verified by confocal microscopy and flow cytometry. NT4 binding was abolished by heparin in both cell lines, and was also inhibited by the known heparin-binding proteins Midkine and, to a lower extent, Apolipoprotein E4, in line with what we had reported with different human cancer cell lines [16], demonstrating specific recognition of NT4 membrane targets. On the contrary, internalization of MTX, as tested by flow cytometry using a fluorescein-conjugated drug, was lower in MDA-MB 231 than in MCF-7.

We proved that the lower intracellular MTX detected in the MTX-resistant MDA-MB 231 cells was due to lower internalization rather than higher extrusion by ABC transporters. In fact, the two cell lines had comparable efficiency in Rho 123 extrusion.

When we studied expression of all MTX folate transporters and receptors in both cell lines, we found that MDA-MB 231 did not express RFC, the inactivating mutations or deficient expression of which is known to be associated with cancer cell resistance to antifolates. This was not surprising since the MDA-MB 231 cell line was already known to be resistant to MTX due to lower expression of RFC [38, 39].

Since the defect in MTX internalization in MDA-MB 231 is due to lack of expression of the main folate transporter, we investigated whether we could by-pass MTX resistance in these cells by switching MTX internalization to a completely different receptor-mediated mechanism, allowed by coupling MTX to NT4 peptides. The regiochemistry of conjugation did not prove to be a discriminant for overall cytotoxic activity and indeed the cytotoxicity of NT4-MTX was very similar in the two cell lines, with the MTX resistant cell line MDA-MB 231 becoming even more sensitive to NT4-MTX compared to the MTX sensitive MCF-7 cell line.

The EC50 of NT4-MTX is in the range of 10^−7^ M in both MTX sensitive MCF-7 and MTX resistant MDA-MB 231 cell lines. This value is about ten times lower than that of the free drug in MCF-7 cells and is almost identical to the EC50 of free MTX in MDA-MB 231. Although apparently disappointing, this result is in line with what we reported in previous papers on NT4-MTX *in vitro* cytotoxicity on different cell lines [12, 14] and also on *in vitro* cytotoxicity of NT4 conjugated with different drugs [11, 14]. In any case, when the same drug-armed NT4 peptides were tested *in vivo* in different animal models of human cancers, they resulted much more efficient than the correspondent unconjugated drug in inhibiting tumor growth or even inducing regression up to clearance of the disease [10, 12, 15]. We are convinced that MTX, when conjugated to the NT4 peptide carriers, is internalized with a different mechanism which depends on NT4 membrane receptors, and this causes the drop in EC50 of MTX as well as of different chemotherapy drugs. This switch to an NT4-mediated drug internalization brings cytotoxicity to be the same in sensitive and resistant cells. This is an advantage, and has to be considered together with the ability of NT4-conjugated drugs to be more potent than the free drug in animal models of human cancers, where we obtained reduction of tumor growth of 60%, 50% and even regression of tumor, with NT4-MTX, NT4-5Fluorodeoxiuridine and NT4-Paclitaxel, respectively [10, 12, 15]. With all the drug-armed NT4 we tested, the cytotoxic activity was lower than that of the free drug, when tested against tumor cells grown *in vitro*, but became higher when the same tumor cells were grafted in mice. We believe that this reproducible result is due to the selective binding of NT4 to cancer cells, which cannot be appreciated *in vitro*, where only cancer cells are present. We already knew then, that conjugation to NT4 can modify the drug biodistribution providing an enhanced tumor accumulation and therefore good efficacy results. Now we can also say that conjugation of a chemotherapy drug to NT4, by allowing a different and drug-independent cell binding and internalization, can bring to the advantage of bypassing drug-resistance of cancer cells.

In conclusion, NT4 peptides seem to be extremely promising cancer-selective targeting agents that can be exploited as theranostics in personalized oncological applications. The present results show that NT4 peptides can overcome drug resistance, which is a big advantage for their application in oncology.

## MATERIALS AND METHODS

### Peptide synthesis

Peptides were synthesized on an automated multiple synthesizer (MultiSynTech, Germany) by standard Fmoc chemistry. Protected L-amino acids and Tentagel-resin were purchased from Iris Biotech, Germany, DIPEA (N,N-diisopropylethylamine) from Merck and HBTU (O-benzotriazole-N,N,N’,N’-tetramethyl-uronium-hexafluoro-phosphate) from MultiSynTech. Coupling times, 1.5 h rt; Fmoc deprotection times: 20 min, rt.

NT4-biotin was synthesized on Tentagel resin with Fmoc-Lys(biotin)-OH as first coupling step, and Fmoc-PEG12-OH as second; two coupling steps with Fmoc-Lys(Fmoc)-OH were then used to build the tetrameric core. Pyro-Glu-O-pentachlorophenylester (Bachem, Switzerland) was used for the last coupling step, since pyro-Glu is the N-terminal acid of the neurotensin sequence.

Peptide conjugated with methotrexate was synthesized using Fmoc-Lys(Dde)-OH as the first amino acid on Tentagel resin and Fmoc-βAla-OH as the second amino acid. Two coupling steps with Fmoc-Lys(Fmoc)-OH were used to build the core. Pyro-Glu-O-pentachlorophenylester was the N-terminal acid. Once the sequence was completed, the Dde protective group was removed using 2% hydrazine in DMF (v/v) on resin and the free amino group was coupled with Fmoc–PEG12–OH. The Fmoc group was then removed to enable coupling with Fmoc-L-Glu(tBu)-OH in the case of 1 and with Fmoc-L-Glu-OtBu in the case of 2. Finally Fmoc was removed and 4-[N-(2,4-diamino-6-pteridinylmethyl)-N-methylamino]benzoic acid was conjugated using HBTU and DIPEA as coupling reagents. Once solid-phase synthesis was completed, the peptides were cleaved from the resin, deprotected and lyophilized.

HPLC purification was performed on a C18 Jupiter Phenomenex column (300 Å, 250 × 10 mm). Water (A) containing 0.1% TFA and acetonitrile (B) were used as eluents in a linear gradient, from 75% to 65% of A in 40 min at flow rate of 4 ml/min. The compounds were characterized on MALDI-TOF mass spectrometer (Ultraflex III Bruker Daltonics).

1, NT4-α-MTX, C_386_ H_604_ N_102_ O_99_. MALDI-MS: 8259,62 [M+H]+. RP-HPLC: t_R_ = 18,80 min, purity > 99%.

2, NT4-γ-MTX, C_386_ H_604_ N_102_ O_99_. MALDI-MS: 8253,81 [M+H]+. RP-HPLC: t_R_ = 19,80 min, purity > 99%.

### Cell cultures

MCF-7 human breast adenocarcinoma, MDA-MB 231 human breast adenocarcinoma, T-24 human bladder carcinoma, PANC-1 human pancreatic carcinoma, HT29 human colon adenocarcinoma, A375 human melanoma, HT-1376 human bladder carcinoma and OVCAR-3 human ovarian carcinoma cells were grown in their recommended media supplemented with 10% fetal calf serum, 200 μg/ml glutamine, 100 μg/ml streptomycin, 60 μg/ml penicillin, and maintained at 37°C, 5% CO_2_. MDA-MB 231 was maintained at 37°C without CO_2_. Cell lines were purchased from ATCC.

### Cytotoxicity

MCF-7 and MDA-MB 231 cells were plated at a density of 5 × 10^3^ per well in 96-well microplates. Different concentrations of NT4-α-MTX, NT4-γ-MTX, MTX or NT4 were added 24 h after plating and cells were incubated for 6 days at 37°C. For the cytotoxicity assay with heparin, cells were exposed to different concentration of NT4-α-MTX or MTX, from 610 pM to 10 μM, in the presence of 1 μM heparin. Cell growth was analyzed after 6 days of incubation at 37°C. Growth inhibition was assessed by 3-(4,5-dimethylthiazol-2-yl)-2,5-diphenyltetrazolium bromide (MTT). Cytotoxicity of drug-conjugated NT4 was compared with that of the corresponding free drugs. The experiment was performed twice in triplicate. EC50 values were calculated by non-linear regression analysis using Graph Pad Prism 5.03 software. Values from untreated controls gave 100% cell viability.

### Immunofluorescence

5 × 10^4^ cells/well MCF-7 and MDA-MB 231 were seeded on 24-well plates, grown for 24 hours, and then incubated with NT4-biotin for 30 min at 37°C (2 μM in PBS-1% BSA), followed by incubation for 30 minutes with 0.5 μg/ml Streptavidin-Atto 550 at room temperature. The cells were then washed and grown in medium for 1, 2 or 4 h at 37°C to allow peptide internalization. They were then fixed with 4% formalin in PBS and plasma membranes were stained with wheat germ agglutinin, Alexa Fluor 647 conjugate (2.5 μg/mL in TBS-1% BSA) incubated for 10 min at room temperature and the nuclei were stained with DAPI (0.5 μg/ml in TBS-1% BSA). Each step was followed by three washes in PBS. Peptide binding was analyzed by confocal laser microscope (Leica TCS SP5) with 380 λ ex and 450-470 λ em, 550 λ ex and 570-590 λ em, 633 λ ex and 660-680 λ em for DAPI, Atto 550 and Atto 647, respectively.

### Flow cytometry

All experiments were performed using 2 × 10^5^ MCF-7 or MDA-MB 231 cells in 96 well U-bottom plates. All dilutions were performed in PBS, containing 5 mM EDTA and 0.5% BSA. 10,000 events were evaluated in a BD FACS Canto II or a BD FACS Calibur (Becton Dickinson, NJ USA). The results were analyzed by FCS Express 6 Flow cytometry software.

Inhibition of NT4 binding by heparin was carried out incubating cells with 250 nM biotinylated NT4 and various concentrations (20 μg/ml, 10 μg/ml and 1 μg/ml) of heparin for 30 min at room temperature. Inhibition of NT4 binding by heparin binding proteins was carried out incubating cells with 250 nM biotinylated NT4 and 1 μM Midkine or Apolipoprotein E4 for 30 min at room temperature. Cells were finally incubated with 1 μg/ml Streptavidin-FITC.

Internalization of fluo-MTX was carried out incubating cells with 10 μM fluo-MTX for 15, 30 and 60 min at room temperature.

To analyze active cell drug export mechanisms, MCF-7 and MDA-MB 231 cells were exposed to 1 μM Rho 123 for 10 minutes, then washed twice with PBS-5 mM EDTA-5% BSA to remove excess of Rho 123, and fluorescence was monitored immediately (T0) and after 10, 20, 30, 45, 60, 90 and 120 minutes. Each step was followed by three washes in PBS-5 mM EDTA-0.5% BSA. *P* values were calculated using two-tailed Student's *t*-test (99% confidence interval) using Graph Pad Prism 5.03 software. *P* values were reported in figure legend.

### Gene expression

Total RNA from 1 × 10^6^ MCF-7 and MDA-MB 231 cells was extracted using an RNeasy kit (QIAGEN, Germantown, MD, US) according to the manufacturer's instructions. One step RT-PCR (QIAGEN) was applied for retrotranscription and human cDNA amplification of RFC (636 pb), PCFT (569 pb), FR-α (568 pb), FR-β (439 pb), FR-γ (400 pb) and GAPSH (207 pb). Human GAPDH was used as reference gene. The following oligonucleotides were used as primers:

RFC primers were 5′-TGAATTCCTGAGC CCAGTGACAAC-3′ (sense), 5′AGCACCAAGGATGAC CAGCAAT-3′ (antisense); PCFT primers were 5′-ACTC TACCCAGCCACTCTGAACTT-3′ (sense), 5′-GATGA CTGCTACACGTGTTGGGAA-3′ (antisense); FR- α primers were 5′-CCTGCAAACGGCATTTCATCCA-3′ (sense), 5′-GGAGCAGGAACCAAATAGTTGGGA-3′ (antisense); FR-β primers were 5′-CCTGGATGTGCC CTTATGCAAAGA-3′ (sense), 5′-TTATCCAAGCTGAG GGCAGGTAGT-3′ (antisense); FR-γ primers were 5′-GA CCTGCTCAATGTCTGCATGAAC-3′ (sense), 5′-CT GAGGTCCAATTCCAGCCTTTGT-3′ (antisense); GAPDH primers were 5′-TCCTCTGACTTCA ACAGCGACACC-3′ (sense), 5′-TCTCTCTTCCTCTTGT GCTCTTGG-3′ (antisense). The following PCR conditions were applied: for RFC 30 cycles of denaturation at 94°C for 60 s, annealing at 58°C for 60 s and extension at 72°C for 90 s; for PCFT, FR-α and FR-β 30 cycles of denaturation at 94°C for 60 s, annealing at 57°C for 60 s and extension at 72°C for 90 s; for FR-γ 30 cycles of denaturation at 94°C for 60 s, annealing at 60°C for 60 s and extension at 72°C for 90 s; for GAPDH 28 cycles of denaturation at 94°C for 30 s, annealing at 60°C for 30 s and extension at 72°C for 30 s.

## Abbreviations

MTX: methotrexate
RFC: reduced folate carrier
PCFT: proton-coupled folate transporter
FR: folate receptor
DHFR: dihydrofolate reductase
TS: thymidylate synthase
GAG: glycosaminoglycans
HSPG: heparan sulfate proteoglycans
LDLR: low density lipoprotein receptor
LRP: lipoprotein receptor-related proteins
fluo: fluorescein
Rho123: rhodamine 123.
